# A piggyBac-based TANGO GFP assay for high throughput screening of GPCR ligands in live cells

**DOI:** 10.1186/s12964-019-0359-x

**Published:** 2019-05-23

**Authors:** Fei Li, Xi Jiang, Ling-Ling Luo, Yue-Ming Xu, Xing-Xu Huang, Cheng Huang, Yu Zhang

**Affiliations:** 1 School of Life Science and Technology, ShanghaiTech University, Shanghai, China; 2 iHuman Institute, ShanghaiTech University, Shanghai, China; 3 School of Pharmacy, Shanghai University of Traditional Chinese Medicine, Shanghai, China

**Keywords:** GPCRs, Ligand piggyBac-TANGO, High signal-to-background ratio, Stable readout, Live cells

## Abstract

**Background:**

GPCRs are considered essential for various physiological processes and have been the most productive drug targets. Therefore, development of the methods of GPCR ligands screening is a high priority for pharmaceutical industries and research institutions.

**Methods:**

We developed a potential method (piggyBac-TANGO) based on the TANGO and PRESTO-TANGO assays. The system was optimized with a piggyBac transposon as a transgene vehicle, and eGFP was used as a reporter instead of luciferase. The assay was validated in the HEK 293T and U87-MG cell lines and antagonist activities of the compounds were assessed. The transgene copy number and long-term stability were evaluated by qPCR. Then, we performed a DRD2-targeted screening for natural products using the piggyBac-TANGO assay.

**Results:**

The validation assay showed that using the piggyBac transposon as a transgene vehicle produced high signal-to-background ratio and stable readout confirmed by investigation of the transgene copy number and long-term stability. Use of eGFP instead of luciferase as a reporter enabled to create a high throughput system suitable for live cells. Moreover, the piggyBac-TANGO assay permitted versatile detection of antagonist activity of compounds and was not limited to a particular cell type. With the use of the piggyBac-TANGO assay, we have successfully identified a novel agonist of DRD2.

**Conclusion:**

Thus, the results indicate that the piggyBac-TANGO method is a user-friendly, robust and imaging-based assay that provides a novel approach to high throughput GPCR-targeted ligand screening and drug development.

**Electronic supplementary material:**

The online version of this article (10.1186/s12964-019-0359-x) contains supplementary material, which is available to authorized users.

## Background

GPCRs are seven transmembrane domain integral membrane proteins that form the largest family of the cell surface receptors capable of transmitting extracellular stimuli into intracellular signals. The extracellular stimuli are physically and chemically diverse and include light, lipids, ions, peptide and non-peptide hormones, neurotransmitters, proteins, growth factors and natural products [1–4]. GPCR activation is involved in a wide variety of physiological processes, such as cell proliferation, survival, differentiation, development, cell-cell communication, and neuronal and hormonal signalling [5, 6]. Critical functions of GPCRs in human pathophysiology and their pharmacological tractability make GPCRs the most popular druggable targets. As of 2017, GPCRs are the targets of 34% (475) of Food and Drug Administration (FDA)-approved drugs with annual profits over 180 billion US dollars [7–9]. Thus, the GPCR ligand screening and method development for identification of the ligands remain the major area of interest for pharmaceutical industries and research institutions.

In the past few decades, the most common assays used for GRCR ligand screening were based on the G-protein function including GTPγS-binding, cAMP and IP_3_/IP_1_ and Ca^2+^ assays [10–14]. However, G-protein-dependent functional assays are not suitable for orphan GPCRs and universal screening due to limitations of G-protein types. β-arrestin-based assays are the G-protein-independent functional assays that are universal and feasible for almost all GPCRs [15, 16]. Barnea et al. and B.L. Roth et al. developed the TANGO (transcriptional activation following arrestin translocation) and PRESTO-TANGO (parallel receptor-ome expression and screening via transcription output-TANGO) methods based on β-arrestin recruitment and showed their advantages for the GPCR ligand screening including feasibility for almost all GPCRs and detection of antagonist activity[17, 18]. PRESTO-TANGO assay, based on TANGO assay, is suitable for “first-round” screening of compound libraries and for identifying ligand of orphan receptors. However, cell lines used in the TANGO and PRESTO-TANGO assays are generated by traditional transfection and antibiotic selection. These procedures frequently result in a low copy number of the transgenes integrated into the host genome. Moreover, transgene expression often declines during long-term cultivation [17–19]. Additionally, the TANGO and PRESTO-TANGO systems use luciferase as a reporter and luciferase can only be assayed in the dead cells thus limiting the throughput. The piggyBac transposon is a mobile genetic element that efficiently integrates the plasmid DNA into mammalian cells via a “copy and paste” mechanism. When the piggyBac transposase protein is expressed, it can recognize transposon-specific inverted terminal repeat (ITRs) sequences and can insert a transgene into the genome at a TTAA site. The piggyBac transposon is characterized by high efficiency (multigene integration), safety (footprint-free removal) and stability (steady long-term expression) and has been considered as a powerful strategy to generate stable cell lines for drug discovery [17, 18, 20, 21].

In the present study, we used the piggyBac dual vector system to generate a stable HEK 293T cell line expressing the β-arrestin-TEV fusion gene and the tTA-dependent GFP expression plasmid for the TANGO assay. We refer to this method as the piggyBac-TANGO assay. The principle of the piggyBac-TANGO assay is essentially the same as the general concept developed by Barnea et al. and B.L. Roth et al. Certain notable optimizations include the use of the piggyBac transposon as a transgene vehicle and eGFP instead of luciferase as a reporter thus improving the piggyBac-TANGO system due to high signal-to-background ratio, stable readout and ability to perform the whole assay in the live cells. The assay is imaging-based and thus, can be used for high throughput screening and simultaneous detection of cytotoxicity of the compounds. The results indicate that the piggyBac-TANGO assay provides a robust, stable and imaging-based platform for high throughput GPCR-targeted ligand screening in living cells.

## Material and methods

### Materials

Cabergoline was purchased from Abcam (Cambridge, UK). 5′-N-Ethylcarboxamidoadenosine was obtained from Sigma (Darmstadt, Germany). Isoproterenol (hydrochloride) was from Cayman (Ann Arbor, Michigan, USA). Chlorpromazine (hydrochloride) and MBX2982 was obtained from Medchemexpress (Monmouth Junction, NJ, USA). Neuromedin U-25 was purchased from Phoenix pharmaceuticals (Burlingame, CA, USA). α-MSH was obtained from APExBIO (Houston, TX, USA).

### Plasmid constructs

#### PB-arrestin2-TEV protease-T2A-puro

As described previously [22], the coding region of human arrestin 2 was amplified with 5′ Xbal I and 3′ BamH I sites. TEV NIa protease coding sequence was created by PCR to add a linker at the 5′ end and a hemagglutinin (HA) tag and EcoR I restriction site at the 3′ end. The β-arrestin 2-TEV protease fusion gene was inserted into the CD133-CAR-T2A-puro piggyBac transposon vector (gifted from Dr. Zhu [23]) to replace the CD133-CAR part.

#### PB-TRE-eGFP-P2A-hygro

eGFP was generated by PCR to place a Smal I site at the 5′ end and to place a BamH I site at 3′ end. The eGFP fragment was inserted into the TRE-NLS-3XFlag-CAS9-NLS-P2A-hygro piggyBac transposon vector (gifted from Dr. Zhu, unpublished) to replace the NLS-3XFlag-CAS9-NLS part.

All restriction endonucleases used were obtained from New England BioLabs. All constructs were verified by DNA sequencing.

### Generation of stable cell lines

HEK-293T and U87-MG cells were obtained from ATCC (Manassas, Virginia, USA). The cell lines were maintained in DMEM (HyClone, Loga, UT, USA) supplemented with 10% FBS (Gemini, West Sacramento, CA, USA) and 1% penicillin/streptomycin (PS, Gibco, Carlsbad, CA, USA). For generation of stable cell lines, 2 × 10^5^ cells were resuspended in 100 μL of Nucleofector™ solution (Lonza, Visp, Switzerland) and transfected with a combination of 1 μg PB-arrestin2-TEV protease-T2A-Puro, 1 μg PB-TRE-eGFP-P2A-hygro and 1 μg Super piggyBac transposase plasmids (System Biosciences, Palo Alto, CA, USA). Electroporation was performed using the DG-130 (HEK 293T) or DS-126 program (U87-MG) on a 4D-Nucleofector (Lonza, Visp, Switzerland). Immediately after transfection, the cells were transferred into 2 mL prewarmed completed medium in a 6 cm dish. After 3 days, drug selection was performed for two weeks with 2 μg/ml puromycin (Invitrogen, San Diego, CA, USA) and 10 μg/ml hygromycin (Invitrogen, San Diego, CA, USA). HEK 293T cells with stable expression of piggyBac-TANGO (HPT cell lines) and U87-MG cells with stable expression of piggyBac-TANGO(UPT cell lines) were maintained in complete DMEM medium supplemented with 2 μg/ml puromycin and 100 μg/ml hygromycin.

### Validation of the piggyBac-TANGO assay

For validation of the piggyBac-TANGO assay, HPT and UPT cell lines were plated overnight in DMEM supplemented with 10% FBS in poly-L-Lysine (Sigma, Darmstadt, Germany) precoated 96-well cell culture plates at a density of 6000 to 8000 cells per well. On the following day (day 2), HPT and UPT cells were transfected with the PRESTO-TANGO expression plasmids (Addgene, Watertown, MA, USA) using Lipofectamine 2000 (Invitrogen, Carlsbad, CA, USA) and incubated in a fresh medium with 10% FBS. On day 3, the corresponding GPCR agonists were added to each well and the cells were incubated for another 24 h. A Nikon microscope (Eclipse Ti-E, Nikon, Tokyo, JP) was used to visualize the fluorescence of HPT and UPT cells at indicated magnification.

### Split luciferase biosensor cAMP assay for activation of DRD2

The split luciferase complementation assay based on the Glosensor cAMP biosensor technology (Promega, Madison, WI, USA) was performed as described in the PDSP protocol book available online at http://pdspdb.unc.edu/pdspWeb/content/. To determine the DRD2-mediated cAMP production, HEK 293T cells were transfected with DRD2 and the Glosensor cAMP plasmids. After 24 h, the transfected cells were seeded into 96 well plates and maintained in DMEM supplemented with 1% dialyzed FBS. On the day of the assay, the medium was removed and the cells were loaded with 20 μl of 4 mM luciferin in assay buffer for 60 min at 37 °C. Then, cells were incubated for 15 min prior to isoproterenol exposure with indicated concentrations of the compounds prepared in 1 × HBSS (Gibco, Carlsbad, CA, USA) containing 0.1% BSA. The plate was read to determine chemiluminescence. The results were analysed using the GraphPad Prism 6 software (La Jolla, CA, USA).

### Copy number quantification

Single HPT cells were sorted into the wells of a 96-well plate and cultured for 2 weeks. Then, single HPT cells were transfected with the ADORA1-TANGO plasmid (Addgene, Watertown, MA, USA) using Lipofectamine 2000 (Invitrogen, Carlsbad, CA, USA). On the following day, single HPT cells without GFP background were treated with NECA. A clone with GFP response was identified and expanded for quantification of the plasmid copy number. Four clones were seeded in a 12-well plate in triplicate and were harvested after reaching confluence in an alkaline lysis buffer (0.2 M NaOH/ 1 mM EDTA) at 55 °C overnight. Genomic DNA was extracted by phenol-chloroform followed by ethanol precipitation. RT-qPCR was carried out in an ABI ViiA 7 system (Life, Carlsbad, CA, USA) with a ChamQ SYBR qPCR master mix (low ROX premixed) (Vazyme, Nanjing, China) according to the manufacturer’s instructions. The forward and reverse primers for eGFP were 5′-ACGACGGCAACTACAAGACC-3′ and 5′-TTGTACTCCAGCTTGTGCCC-3′, respectively. The forward and reverse oligonucleotide primers for GAPDH were 5′-CATCAATGGAAATCCCATCA-3′ and 5′-TTCTCCATGGTGGTGAAGAC-3′, respectively. qPCR was performed with a preincubation cycle at 95 °C for 10 min followed by 40 cycles of amplification (15 s at 95 °C, 60 s at 60 °C, and 15 s at 95 °C). The copy number of eGFP was determined by the standard curves containing wild type HEK 293T genomic DNA (10 ng/μl final concentration) with a serial dilution of a known amounts of the PB-TRE-eGFP-P2A-hygro plasmid as described previously [24].

For investigation of stability, 4 cloned cell lines were maintained in DMEM supplemented with 10% FBS, 1% PS and 2 μg/ml puromycin. The cells were cultured for up to 7 weeks and genomic DNA was collected every week to determine the plasmid copy number. The plasmid copy number quantification was performed as described in the preceding paragraph.

### DRD2-targeted natural product screening

For preliminary screening, the expression plasmid for DRD2-TANGO was transfected into the HPT cells using Lipofectamine 2000. After 24 h, a DRD2 agonist (cabergoline) and natural products were added to the fresh medium and the cells were incubated for another 24 h. Natural product library was obtained from Shanghai R&D Center for Standardization of Chinese Medicines. The final concentrations of cabergoline and the compounds were 10 μM and 25 μM, respectively. The results were visualized using a Nikon microscope at indicated magnification.

## Results

### Design and validation of the piggyBac-TANGO assay

**Fig. 1:**
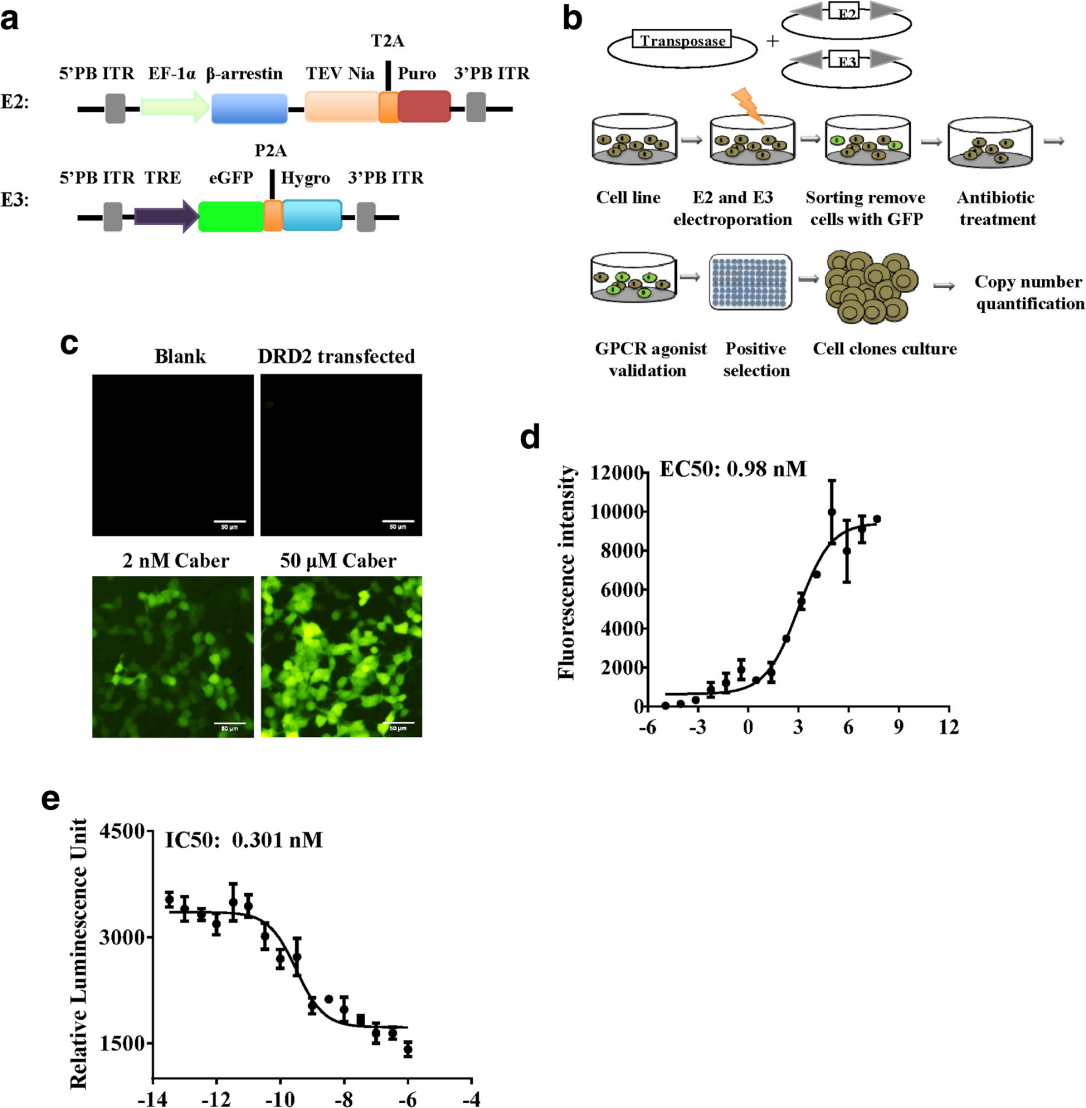
Design and validation of the piggyBac-TANGO assays. **a** Schematic overview of the expression vectors used throughout this study. b Scheme of generation of stable cell lines expressing piggyBac-TANGO. **c** Validation of selected receptors in the piggyBac-TANGO assay. **d** Fluorescence intensity of DRD2 stimulated by cabergoline in the piggyBac-TANGO assay. **e** Concentration-response curves of DRD2 stimulated by cabergoline in the cAMP assay. Data are presented as the means ± SEM (*n* = 3). Caber: Cabergoline. Curves were fitted using GraphPad Prism 6

GPCR screening technologies based on G-protein-independent β-arrestin recruitment assay have many advantages and are suitable for the majority of GPCRs [22, 25]. To develop a method for screening of the GPCR-targeting drugs characterized by a high signal-to-background ratio, stability and imaging-based detection in the live cells, we designed an optimized strategy for the TANGO assay to improve signal-to-background ratio and stability. The arrestin fusion gene and the tTA-dependent reporter gene were incorporated into the piggyBac transfer vectors and eGFP was used as a reporter instead of luciferase (Fig. 1a). This approach is referred as the piggyBac-TANGO assay. Cell lines with stable expression of piggyBac-TANGO were created by electroporation of the parental cell lines with a combination of the PB-arrestin2-TEV protease-T2A-Puro, PB-TRE-eGFP-P2A-hygro and Super piggyBac transposase plasmids. The cells with eGFP background were removed by sorting and the remaining cells were selected for 4 weeks (Fig. 1b). To validate the utility of this system, we transfected HEK 293T cells, which had stable expression of piggyBac-TANGO (HPT cells), with DRD2 and treated the cells with an agonist of DRD2, cabergoline. Figure 1c shows that transfection of the control cells produced no or little fluorescent signal similar to the blank group. However, cabergoline treatment induced a dramatic fluorescence response even at very low concentrations of the agonist. Concentration-response curves and fluorescence images are shown in Fig. 1d and Additional file 1: Figure S1. Cabergoline-induced activation resulted in a 274-fold signal-to-background ratio compared to that in the group without agonist treatment, which is much higher than that of PRESTO-TANGO assay (signal-to-background ratio: 68-fold) [26]. Then, the cAMP concentration assay was performed to confirm cabergoline-induced activation of DRD2 (Fig. 1e). The results indicated that the piggyBac-TANGO assay can indeed be used to test activation of GPCR with high signal-to-background ratio and imaging readout.

### Adaptability of the piggyBac-TANGO assay

**Fig. 2:**
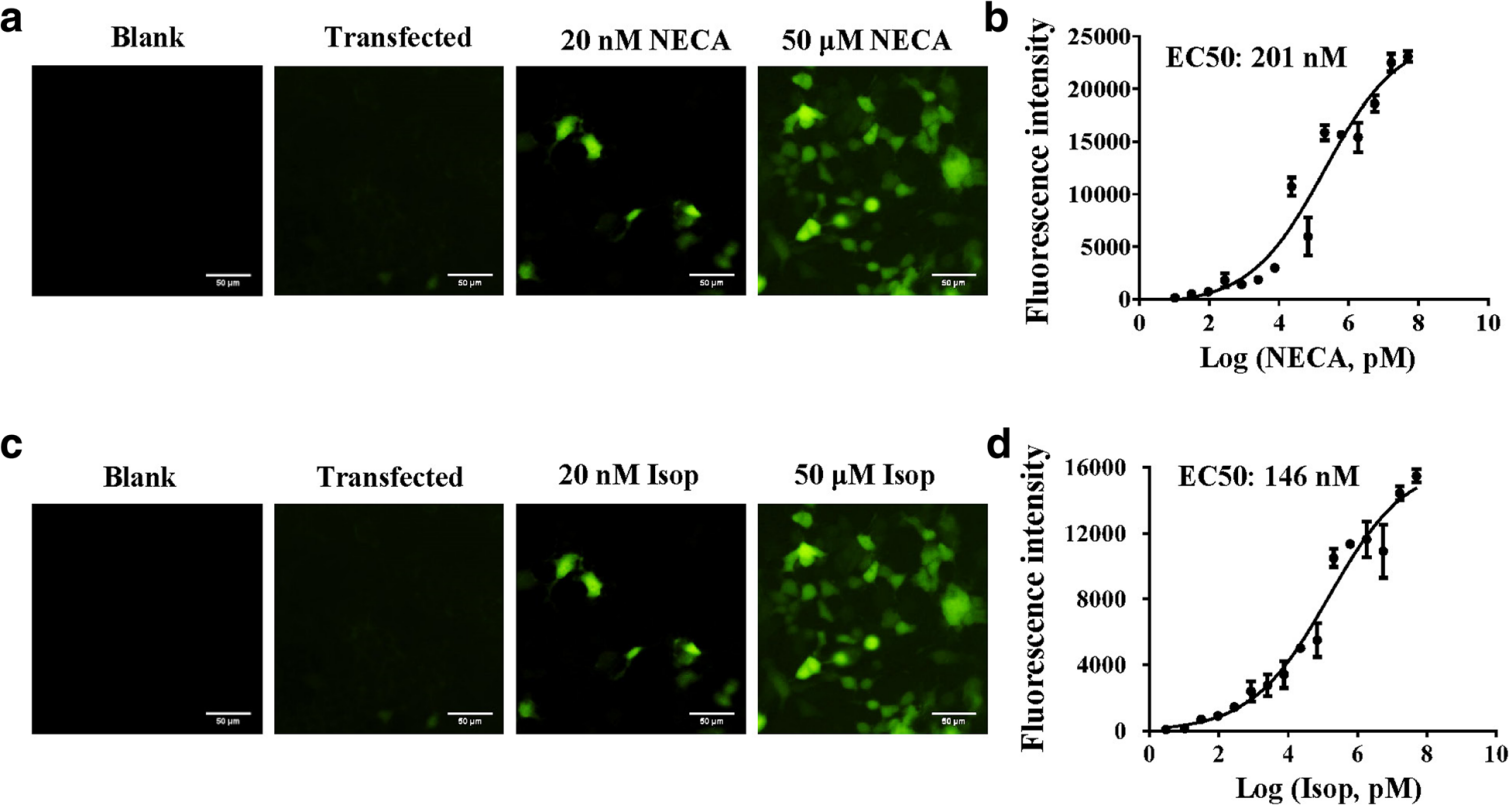
The piggyBac-TANGO assays of adenosine A1 receptor (ADORA1) and β1 adrenergic receptor (ADRB1). **a** NECA induced fluorescence response of ADORA1 in the piggyBac-TANGO assay. **b** Concentration-response curves of ADORA1 stimulated by NECA in the piggyBac-TANGO assay. **c** Isoproterenol-induced fluorescence response of ADRB1 in the piggyBac-TANGO assay. **d** Concentration-response curves of ADRB1 stimulated by isoproterenol in the piggyBac-TANGO assay. Data are presented as the means ± SEM (*n* = 3). NECA, 5′-N-ethylcarboxamidoadenosine; Isop, isoproterenol. Curves were fitted using GraphPad Prism 6

**Fig. 3:**
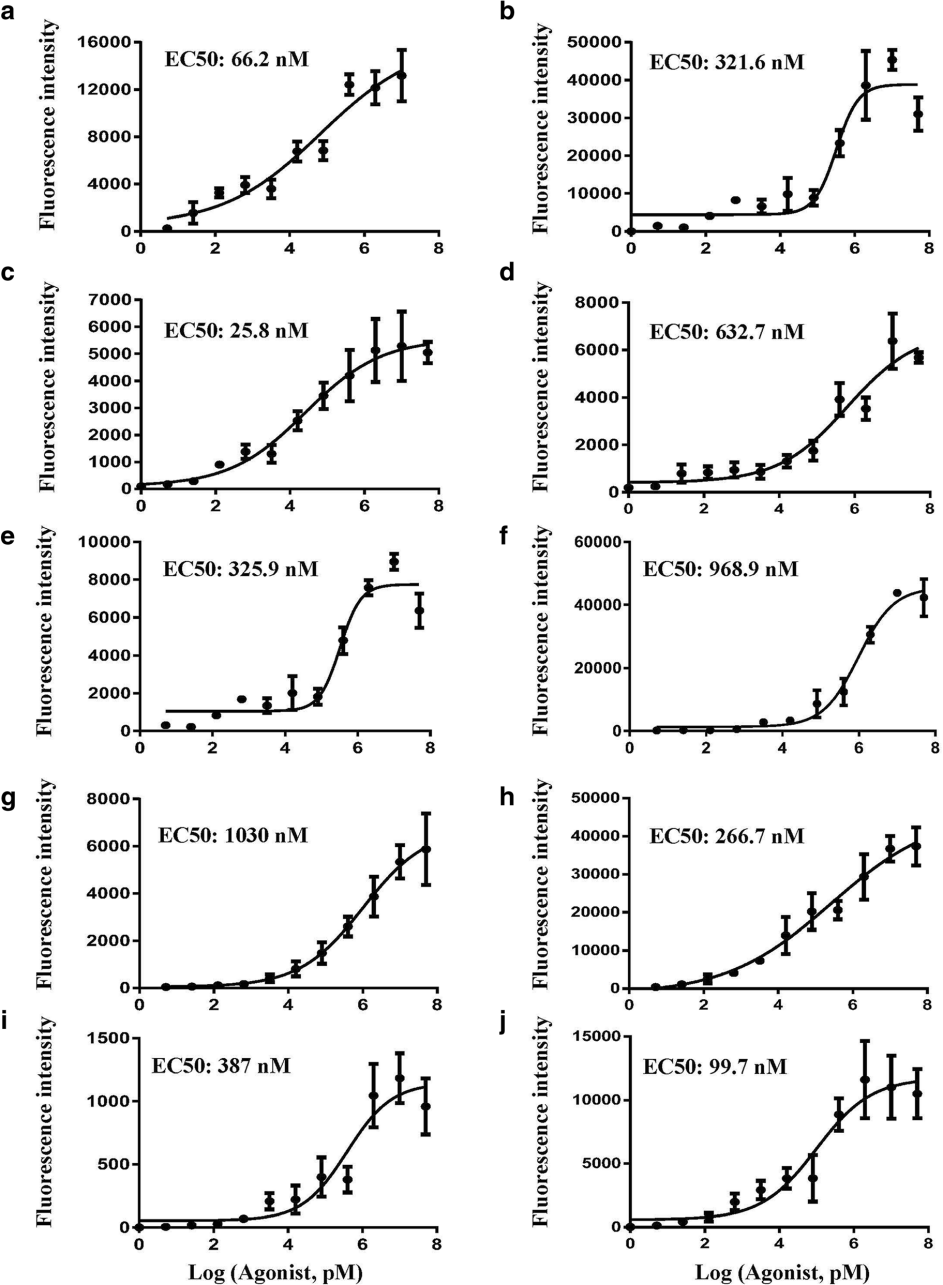
Dose-response curves for the activation of the GPCRs. **a-e** Validation of selected receptors (GPR119, ADRB2, ADRB3, CB1 and DRD1) in the piggyBac-TANGO assay. The agonists of receptors were receptively MBX2982, isoproterenol (for ADRB2 and ADRB3), AM1202(CB1) and cabergoline. **f-j** Validation of selected receptors (DRD3, DRD5, MC4R, NUMR1 and NUMR2) in the piggyBac-TANGO assay. The agonists of receptors were receptively cabergoline (for DRD3 and DRD5), α-MSH(MC4R) and Neuromedin U-25 (for NUMR1 and NUMR2). Data are presented as the means ± SEM (*n* = 3). Curves were fitted using GraphPad Prism 6

To investigate the universality and applicability of the piggyBac-TANGO assay, we tested the ligand-binding activity of the adenosine A1 (ADORA1) and β1 adrenergic receptors (ADRB1). The results were characterized by low background and high fluorescence response similar to the DRD2 tests (Fig. 2a and c). Concentration-response curves and fluorescence images are presented in Fig. 2b, Fig. 2d and Additional file 2: Figure S2; the signal-to-background ratios were 182-fold and 175-fold, respectively. Consistent with the result of DRD2, signal-to-background ratios of ADORA1 and ADRB1 are higher than that of PRESTO-TANGO assay (ADORA1: 4.8-fold, ADRB1: 22-fold). Furthermore, ten receptors for activation by their agonists were validated. Similar to above results, agonist-induced activation of TANGO-ized GPCR created high fluorescence response (Fig. 3a-j and Additional file 3: Figure S3). These data suggest that the piggyBac-TANGO assay can measure ligand activity for most of the GPCRs with high signal-to-background ratio.

Additionally, we generated the U87-MG cells that have stable expression of piggyBac-TANGO (UPT cell lines) to test whether the measurement of the ligand-binding activity of GPCR based on the piggyBac-TANGO assay can be performed in other cell lines. As expected, cabergoline and NECA induced fluorescence responses in the case of DRD2 and ADORA1, respectively, suggesting that the piggyBac-TANGO assay has no cell type limitations (Additional file 4: Figure S4).

### Measurement of agonist and antagonist activity using the piggyBac-TANGO assay

**Fig. 4:**
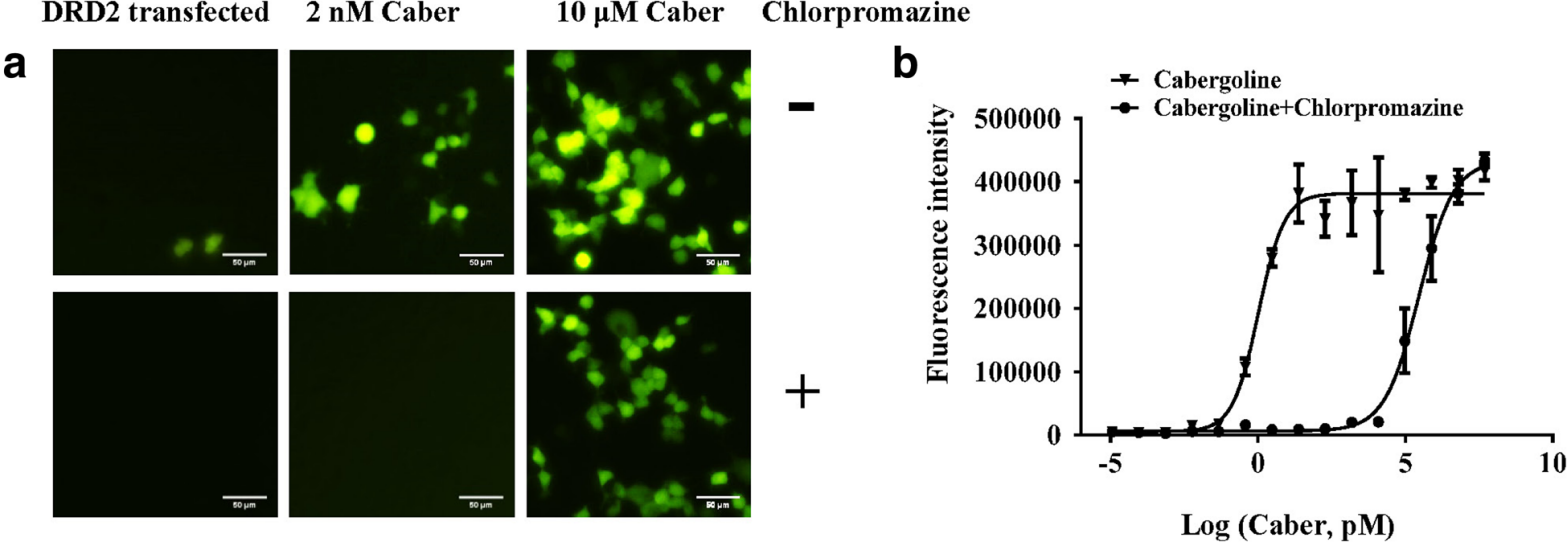
Use of the piggyBac-TANGO assay to profile a DRD2 agonist and an antagonist. We preincubated the DRD2-expressing HPT cells with their potential antagonist chlorpromazine. After 6 h, cabergoline was added into medium for additional 24 h exposure. **a** Fluorescence response of DRD2 induced by cabergoline and chlorpromazine in the piggyBac-TANGO assay. **b** Concentration-response curves of DRD2 stimulated by cabergoline and chlorpromazine in the piggyBac-TANGO assay. Data are presented as the means ± SEM (*n* = 3). Curves were fitted using GraphPad Prism 6

Then, we investigated whether an antagonist activity of a tested compound can be measured using the piggyBac-TANGO assay. We preincubated GPCR-expressing HPT cells with a potential antagonist before exposure to an agonist. The imaging-based results indicated that the fluorescence response recovered concomitant to an increase in the agonist concentration indicating that the activation of DRD2 induced by cabergoline can be blocked by antagonist chlorpromazine (Fig. 4a and Additional file 5: Figure S5). The results showed that the concentration-response curve of cabergoline was shifted to the right by chlorpromazine pre-incubation (Fig. 4b). Thus, these results validate the feasibility of the piggyBac-TANGO assay for determination of antagonist activity.

### Transgene copy number in the piggyBac-TANGO assay

**Fig. 5:**
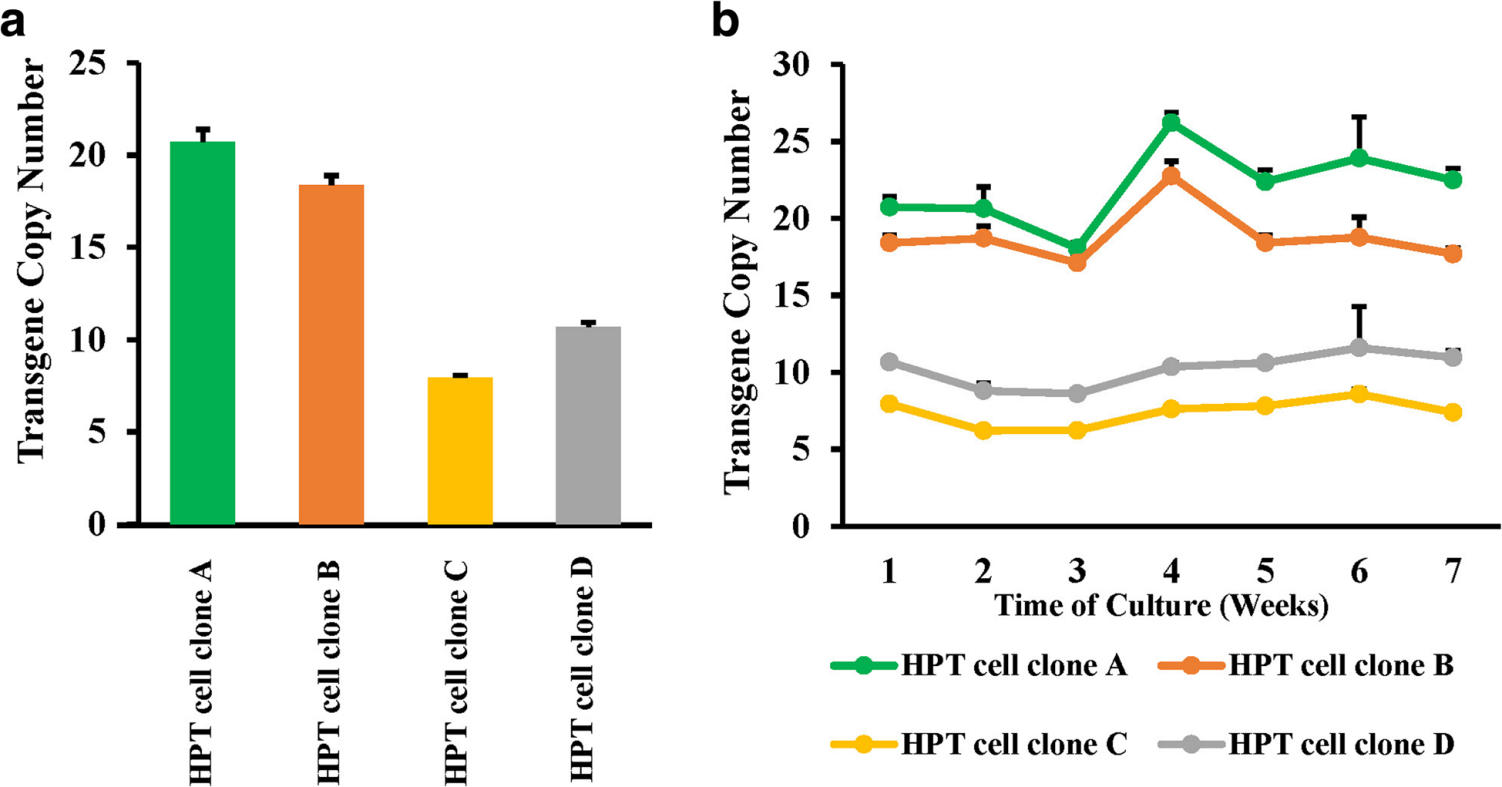
Analysis of the transgene copy number in 4 clonal cell lines. **a** qPCR was performed to measure the eGFP copy number in 4 clonal cell lines. **b** Long-term stability of 4 clonal cell lines. The GAPDH gene was amplified as an internal control to normalize the amount of genomic DNA

The piggyBac transposon can integrate a gene into the host genome at a high copy number [20, 27]. To estimate the copy number of the integrated gene, qPCR was performed to measure eGFP copy number in 4 clonal cell lines. As shown in Fig. 5a, the copy number values of eGFP were 21, 18, 8 and 11 suggesting that a high copy number of the transgenes integrated into the host genome contributes to robustness of the piggyBac-TANGO assay. These results were consistent with previous studies that demonstrated that a PB dual vector system has high efficiency and high copy number of transposition [24, 28].

### Long-term stability of the piggyBac-TANGO assay

To assess the stability of the piggyBac-TANGO assay over time, we collected genomic DNA of 4 cloned cell lines to quantify the transgene copy number every week for 7 weeks. Consistent with our expectations, the copy number of eGFP was not changed significantly in the clones generated by the PB-transposition (Fig. 5b). These results demonstrated the stability of the cell lines generated by the PB-transposition.

### Screening of DRD2-targeting natural products using the piggyBac-TANGO assay

**Fig. 6:**
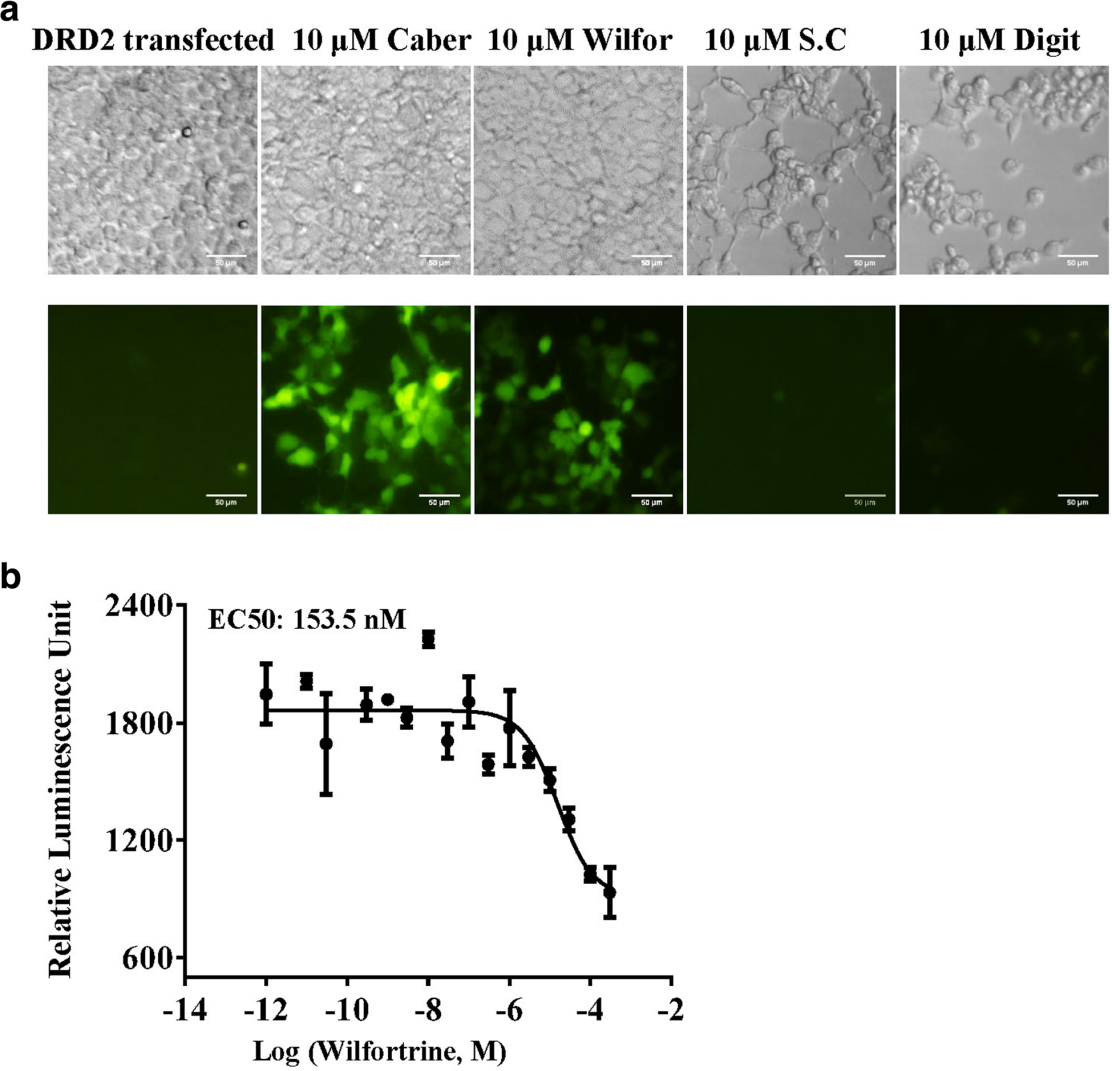
Natural product screening for DRD2-targeting compounds using the piggyBac-TANGO assay. **a** Natural product induced fluorescence response of DRD2 in the piggyBac-TANGO assay. **b** Concentration-response curves of DRD2 stimulated by wilfortrine in the cAMP assay. Data are presented as the means ± SEM (*n* = 3). Caber: Cabergoline; Wilfor: Wilfortrine; S.C: sanguinarine citrate; Digit: digitaline. Curves were fitted using GraphPad Prism 6

Natural products provide an abundant resource for drug discovery. Considering that the piggyBac-TANGO assay is characterized by being robust, user-friendly and imaging-based, the assay is an easy-to-operate high throughput approach for drug development using natural products. Thus, we performed the DRD2-targeted screening of natural products via the piggyBac-TANGO assay. As shown in Fig. 6a, we identified wilfortrine, which was extracted from *Tripterygium Wilfordii* Hook, as a novel agonist of DRD2 from a library of 1000 natural products. Then, the cAMP response assay was performed to confirm the concentration-dependent activity of wilfortrine with regards to DRD2 (Fig. 6b). One of the benefits of cell-based imaging assay is simultaneous detection of compound liabilities. In parallel, we detected cytotoxicity of sanguinarine citrate and digitaline during the imaging-based assay from a library of 1000 natural products (Fig. 6a).

## Discussion

**Fig. 7:**
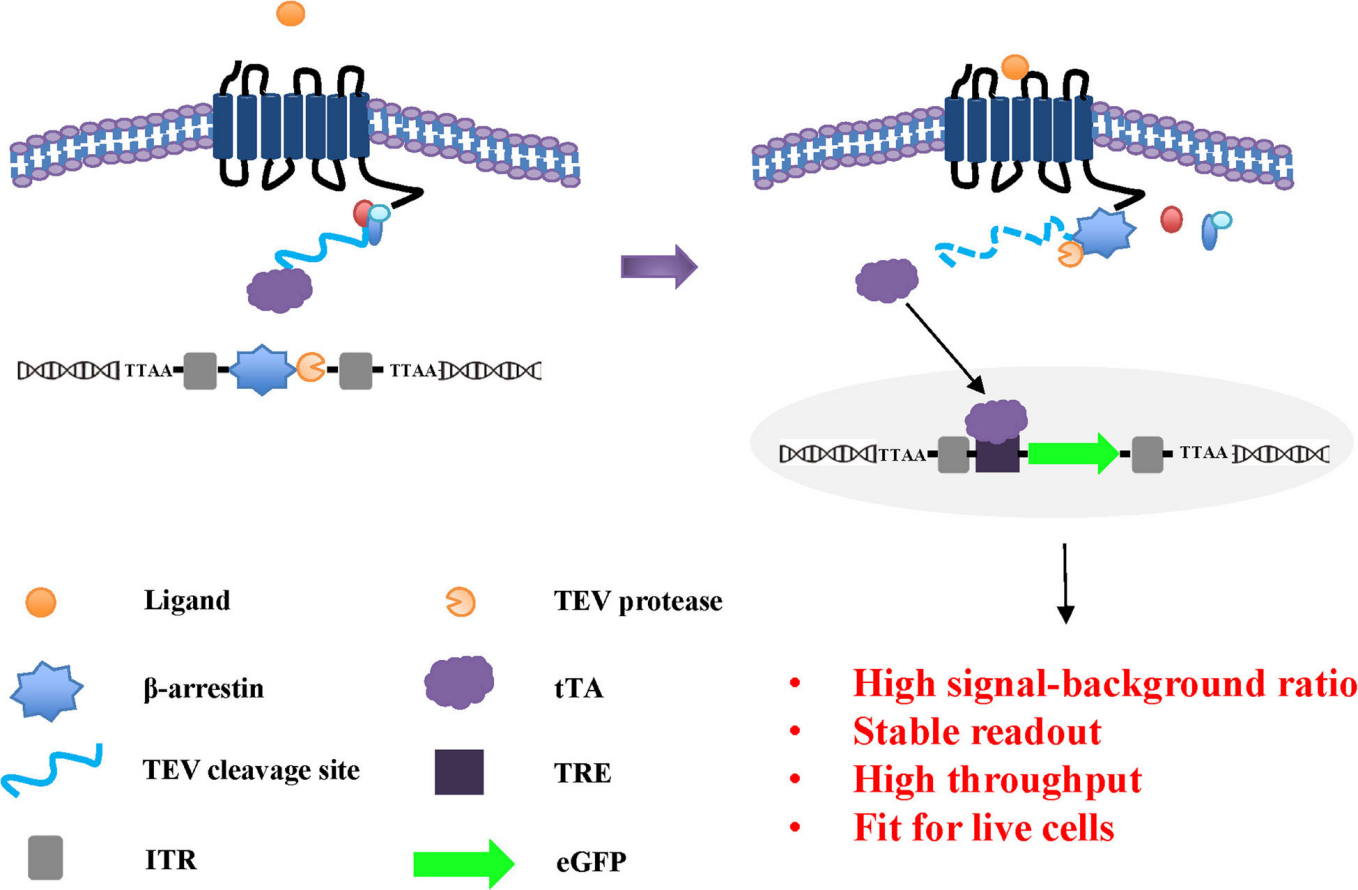
General scheme of piggyBac-TANGO assays. We inserted arrestin fusion genes and tTA-dependent reporter gene into host cell genome at TTAA target site via piggyBac transposon system and choosed eGFP instead of luciferase as reporter gene

In this study, we have developed and validated an imaging-based method named the piggyBac-TANGO assay for GPCR ligand screening in the live cells. As summarized in Fig. 7, piggyBac-TANGO assay was developed by optimization of the TANGO and PRESTO-TANGO assay using the piggyBac transposon system as a DNA delivery vector and GFP as a reporter instead of luciferase. In addition to the strengths of TANGO and PRESTO-TANGO [22, 25], the modifications provide the piggyBac-TANGO with numerous powerful advantages: (i) higher signal-to-background ratio due to high efficiency and multiple copies of the transgene integrated into the host genome; (ii) higher stability without reduction in the transgene expression; (iii) lower cost and lack of fluorescent dyes or secondary substrates; (iv) the cell imaging assay allows for visualization of the cells for parallel determination of putative compound liabilities, such as cytotoxicity. These features make the piggyBac-TANGO assay more suitable for the initial screening of the GPCR ligands.

Barnea et al. and Kroeze et al. have demonstrated several advantages of the TANGO and PRESTO-TANGO assays based on β-arrestin recruitment including activation of the majority of GPCRs and ability to detect antagonist activity [22, 25]. The principle of the piggyBac-TANGO assay is substantially similar to the TANGO and PRESTO-TANGO assay and it should be able to detect activators and antagonists of GPCR. Our study has validated the utility of the piggyBac-TANGO assay for more than ten receptors, such as DRD2, ADRB1 and ADORA1 in the HPT cell lines. Moreover, we observed fluorescence response induced by a GPCR ligand in the UPT cell lines indicating that the piggyBac-TANGO assay is not limited to a particular cell type. Additionally, the piggyBac-TANGO assay, similar to the PRESTO-TANGO assay, was used to determine the activity of a GPCR antagonist. Adaptability to the majority of GPCRs and various cell type and detection of activators and antagonists of GPCR suggest that the piggyBac-TANGO assay is flexible and universal.

Stable cell lines used in the TANGO and PRESTO-TANGO assays are generated by traditional transfection methods. Long time required for generation of a cell line due to low efficiency and multiple rounds of transfection and antibiotic selection is the flaw of the traditional methods [17–19]. Moreover, the copy number of the transgenes inserted into the host genome is low and can be reduced over time [24, 29]. Therefore, the stable cell lines used in the previous studies were not an ideal model for drug development. In our study, stable cell lines were generated using the piggyBac transposon as a DNA delivery vector. The piggyBac transposon system is a new genetic vehicle and an attractive tool for gene delivery into the mammalian genomes due to its high efficiency, safety and stability. The piggyBac system was harnessed to create cell lines for multigene integration requiring only a single transfection followed by a shorter time for antibiotic selection and expansion [21, 27, 30]. Furthermore, the transposon elements are capable of integrating 10 or more exogenous DNA plasmids into a host cell genome and transposition predominantly targets transcriptionally active sites of the host genomes thus resulting in higher transgene expression [28, 31–33]. In agreement with previous studies [28, 31, 32], our analysis of the transgene copy number demonstrated that the cell clones had a high transgene copy number and achieved integration of 10 or more exogenous DNA plasmids thus contributing to high signal-to-background ratios and high readout signals. Stability is another critical factor of an ideal cell model for long-term compound screening. Several studies have reported piggyBac-mediated long-term and stable gene expression in vitro and vivo [21, 34, 35]. In our study, significant changes in the eGFP level were not observed according to the results of quantification of copy number during the seven-week culture. Besides, footprint-free removal that leaves no piggyBac and transposase sequences behind is another important feature for piggyBac system [20], which conduced to safety and steady of piggyBac-TANGO assay. Use of eGFP as a reporter instead of luciferase is another strong point of the piggyBac-TANGO assay. The benefits of this modification include (i) easy operation; (ii) low cost; (iii) visualization of the output; and (iv) simultaneous detection of compound cytotoxicity as reported in previous imaging-based assays [36, 37].

Based on the advantages of the piggyBac-TANGO assay, we have screened DRD2-targeting natural products. The results indicated that the piggyBac-TANGO assay is an easy to operate and high throughput approach for drug development using natural product. Moreover, we successfully identified a novel agonist of DRD2, wilfortrine, and simultaneously determined that it was not cytotoxic within short time. Additionally, we detected that sanguinarine citrate and digitaline have a toxic effect on the cells.

In recent years, the studies about GPCR drug discovery have transferred to discovery of allosteric modulator. As studies were reported, the advantageous characteristics of allosteric modulators are high specificity and low adverse effects compared with orthostertic ligand [38, 39]. The piggyBac-TANGO assay is based on that ligand activation of GPCRs results in the phosphorylation of specific serine and threonine residues by a class of GPCR kinases, thereby recruiting arrestin to prevent further G protein activation. So piggyBac-TANGO assay is particularly suitable for “first-round” screening of compound to distinguish ability of arrestin recruitment or not, which is not capable of identifying allosteric modulator. Further optimization of piggyBac-TANGO assay for allosteric screening is required.

## Conclusions

Thus, we show that the piggyBac transposon is a potential tool to generate a stable cell line for the β-arrestin recruitment assay with certain advantages for long-term screening of the GPCR-targeting compounds. Moreover, use of eGFP as a reporter makes the piggyBac-TANGO assay suitable for imaging-based detection in the live cells. The piggyBac-TANGO assay provides high signal-to-background ratio and represents a more stable and imaging-based approach thus endorsing the use of this platform for improving identification of the GPCR ligands.

## Additional files

Additional file 1: **Figure S1** Concentration-fluorescence response of DRD2 stimulated by Cabergoline in piggyBac-TANGO assay. -: without DRD2 transfection. +: Cabergoline treatment. Number 1-14: the concentration of cabergoline used in piggyBac-TANGO assay corresponds to that of Fig. 1d. (JPG 107 kb)

Additional file 2: **Figure S2** Concentration-fluorescence response of ADORA1 and ADRB1 stimulated by NECA and Isoproterenol in piggyBac-TANGO assay. -: without transfection. +: agonist treatment. Number 1-14: the concentration of agonist used in piggyBac-TANGO assay corresponds to that of Fig. 2b and d. (JPG 202 kb)

Additional file 3: **Figure S3** Concentration-fluorescence response of GPCRs stimulated by their agonist. The agonists are receptively MBX2982 (GPR119), isoproterenol (for ADRB2 and ADRB3), AM1202 (CB1) cabergoline (DRD1, DRD3 and DRD5). α-MSH (MC4R) and Neuromedin U-25 (NUMR1 and NUMR2) in the piggyBac-TANGO assay. (JPG 190 kb)

Additional file 4: **Figure S4** Adaptability of piggyBac-TANGO assays on U-87 cell line. (JPG 59 kb)

Additional file 5: **Figure S5** Concentration-response pictures of DRD2 stimulated by cabergoline and chlorpromazine in the piggyBac-TANGO assay. Number 1-14: the concentration of cabergoline used in piggyBac-TANGO assay corresponds to that of Fig. 4b. (JPG 148 kb)

## Abbreviations

ADORA1: Adenosine A1 receptor
ADRB: β adrenergic receptor
CB1: Cannabinoid receptor type 1
DR: Dopamine receptor
FDA: Food and drug administration
HA: Hemagglutinin
HPT: HEK 293T cells stably expressing piggyBac-TANGO
ITRs: Inverted terminal repeats
MC4R: Melanocortin 4 receptor
NECA: 5′-N-Ethylcarboxamidoadenosine
NMUR: Neuromedin U receptor
PRESTO-TANGO: parallel receptor-ome expression and screening via transcription output-TANGO
PS: Penicillin streptomycin
TANGO: Transcriptional activation following arrestin translocation
UPT: U87-MG cells stably expressing piggyBac-TANGO

## Author’s information

Yu Zhang is a research assistant professor at ShanghaiTech University whose research interests center around development of cell biology approaches, drug development, genome editing and pharmacology. Cheng Huang is a professor at Shanghai University of Traditional Chinese Medicine whose interests focus on drug development and pharmacology. Xing-Xu Huang is a professor at ShanghaiTech University whose research interests focus on genetic and epigenetic changes using genome editing.
