# Supplementary figures and images for: A piggyBac-based TANGO GFP assay for high throughput screening of GPCR ligands in live cells
Source: Cell Commun Signal. 2019 May 23;17:49. doi: 10.1186/s12964-019-0359-x (PMC6533772; doi:10.1186/s12964-019-0359-x)

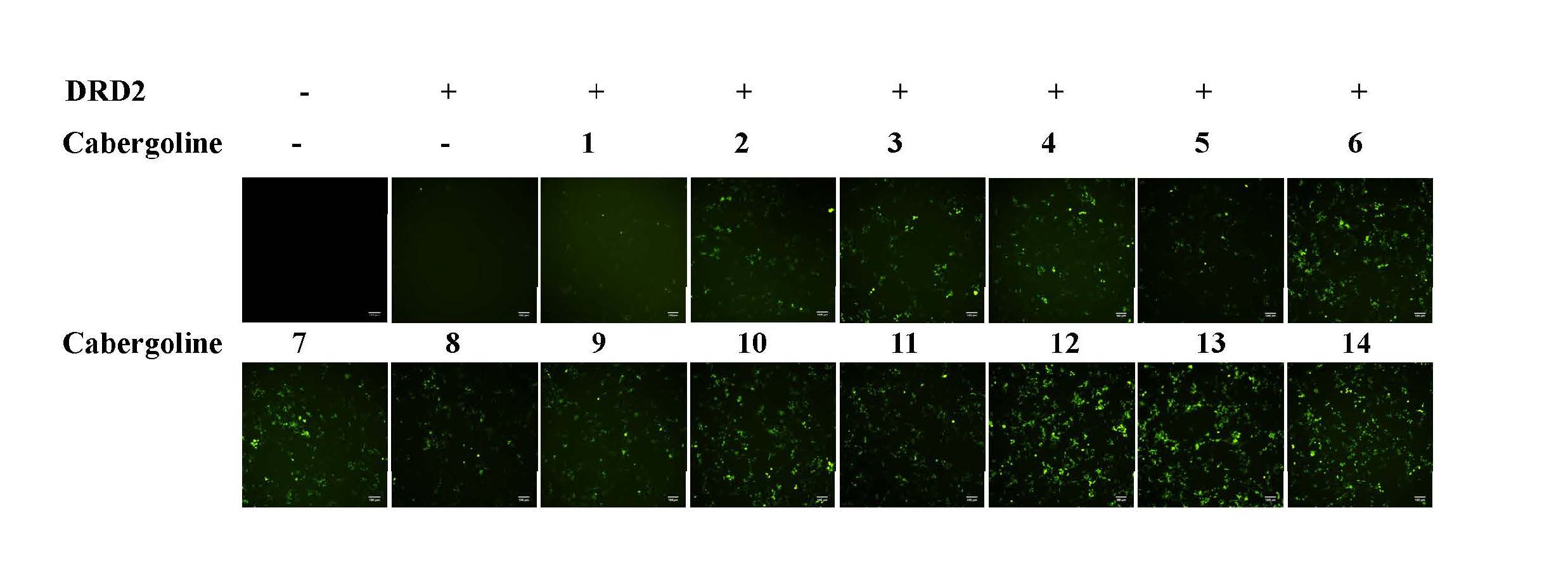

Supplement: Supplementary file 1 — Figure S1 Concentration-fluorescence response of DRD2 stimulated by Cabergoline in piggyBac-TANGO assay. -: without DRD2 transfection. +: Cabergoline treatment. Number 1-14: the concentration of cabergoline used in piggyBac-TANGO assay corresponds to that of Fig. 1d. (JPG 107 kb) [file 12964_2019_359_MOESM1_ESM.jpg]

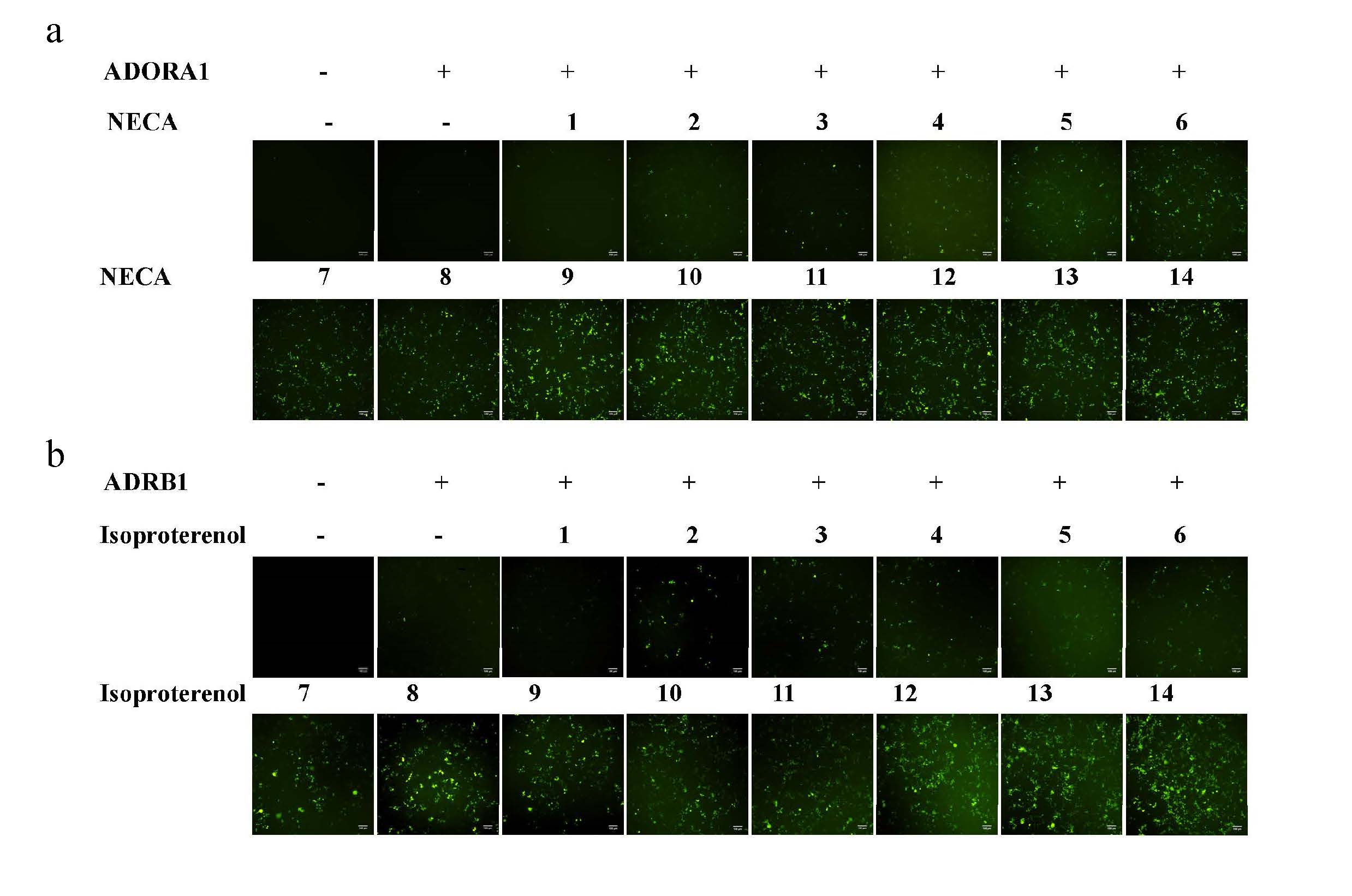

Supplement: Supplementary file 2 — Figure S2 Concentration-fluorescence response of ADORA1 and ADRB1 stimulated by NECA and Isoproterenol in piggyBac-TANGO assay. -: without transfection. +: agonist treatment. Number 1-14: the concentration of agonist used in piggyBac-TANGO assay corresponds to that of Fig. 2b and d. (JPG 202 kb) [file 12964_2019_359_MOESM2_ESM.jpg]

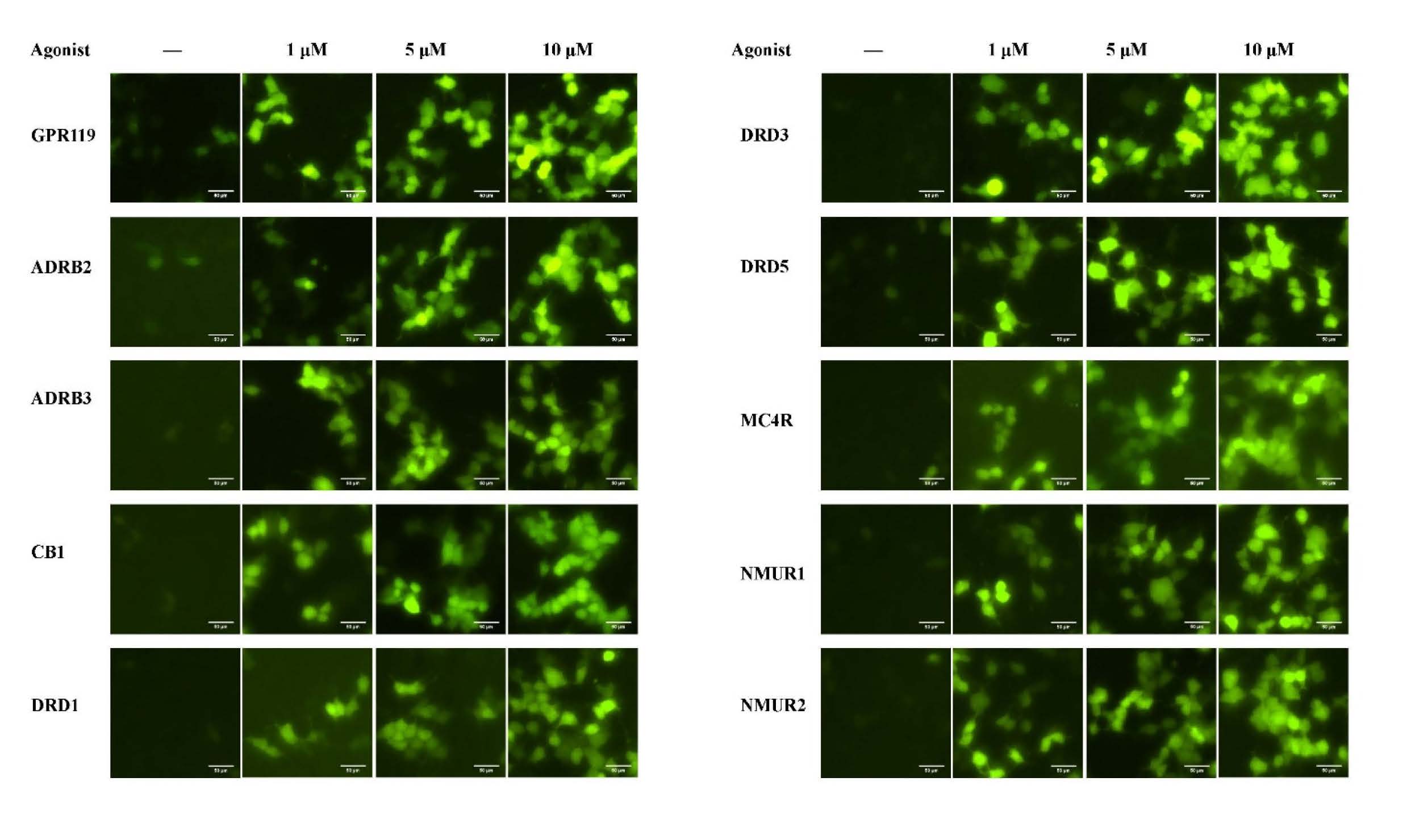

Supplement: Supplementary file 3 — Figure S3 Concentration-fluorescence response of GPCRs stimulated by their agonist. The agonists are receptively MBX2982 (GPR119), isoproterenol (for ADRB2 and ADRB3), AM1202 (CB1) cabergoline (DRD1, DRD3 and DRD5). α-MSH (MC4R) and Neuromedin U-25 (NUMR1 and NUMR2) in the piggyBac-TANGO assay. (JPG 190 kb) [file 12964_2019_359_MOESM3_ESM.jpg]

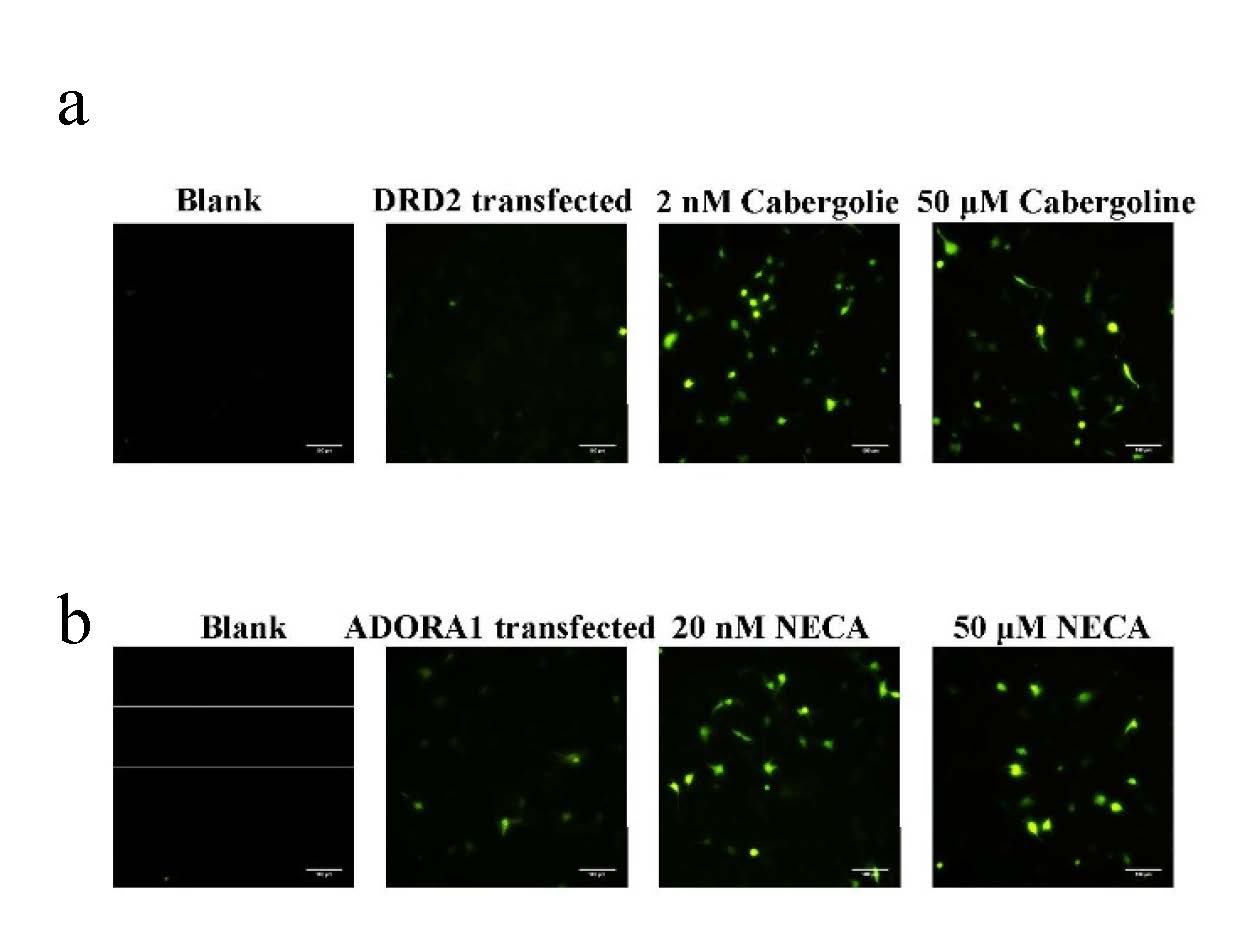

Supplement: Supplementary file 4 — Figure S4 Adaptability of piggyBac-TANGO assays on U-87 cell line. (JPG 59 kb) [file 12964_2019_359_MOESM4_ESM.jpg]

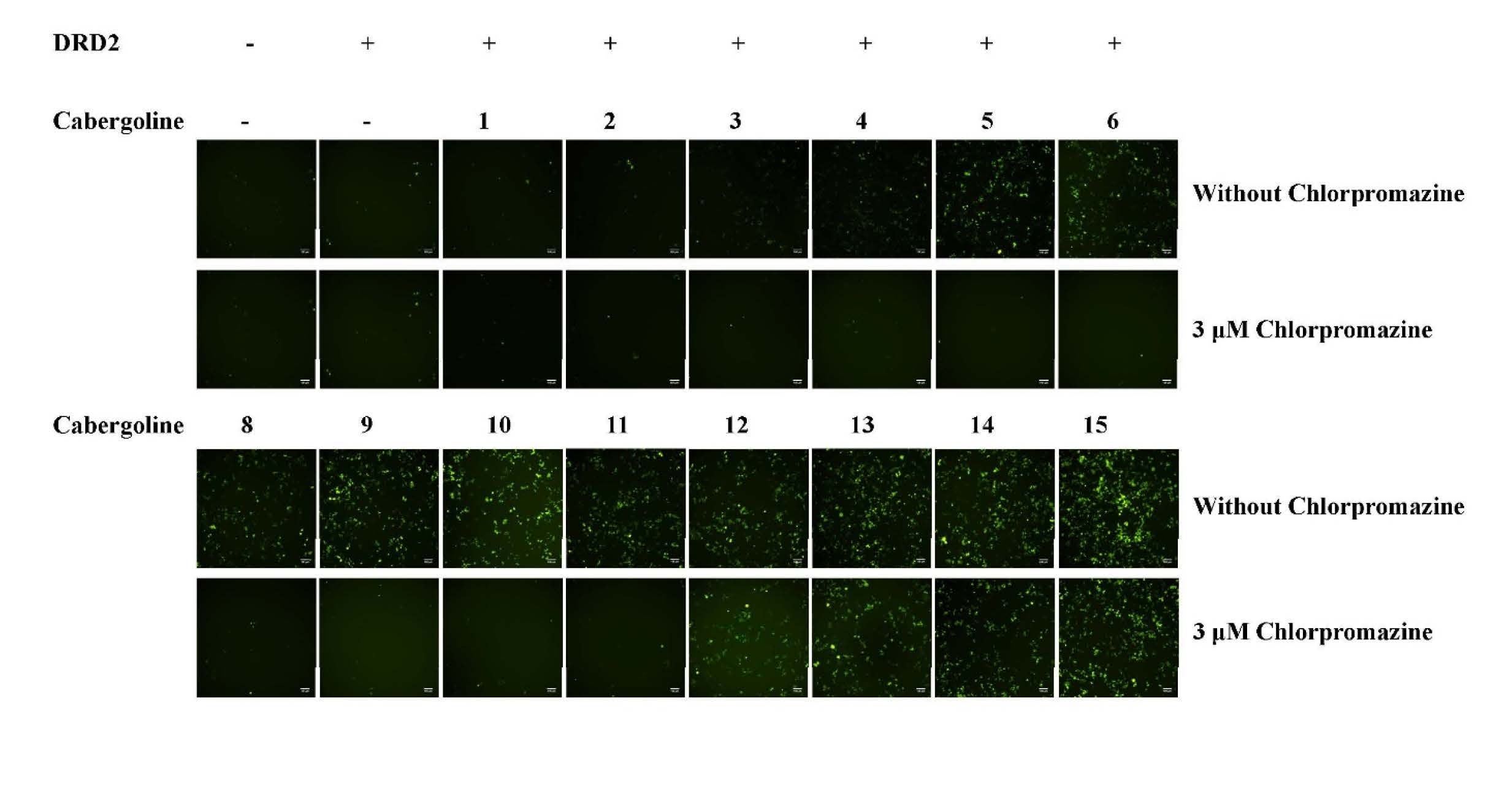

Supplement: Supplementary file 5 — Figure S5 Concentration-response pictures of DRD2 stimulated by cabergoline and chlorpromazine in the piggyBac-TANGO assay. Number 1-14: the concentration of cabergoline used in piggyBac-TANGO assay corresponds to that of Fig. 4b. (JPG 148 kb) [file 12964_2019_359_MOESM5_ESM.jpg]
